# Exploring Risk Factors for Predicting 30-Day Postoperative Morbidity in Musculoskeletal Tumor Surgery

**DOI:** 10.3390/jcm13092681

**Published:** 2024-05-02

**Authors:** Philip Heesen, Maria Elyes, Jan Domanski, Georg Schelling, Sören Könneker, Bruno Fuchs

**Affiliations:** 1 Department of Plastic & Reconstructive Surgery, University Hospital USZ, University of Zurich, 8000 Zurich, Switzerland; 2 Department of Orthopedics & Trauma, Sarcoma Service, LUKS University Hospital, University of Lucerne, 6000 Lucerne, Switzerland; 3 Department of Trauma Surgery and Orthopedics, Ortenau Klinikum, 77654 Offenburg, Germany; 4 Department of Orthopedics and Trauma, Sarcoma Service, Kantonsspital Winterthur, 8400 Winterthur, Switzerland

**Keywords:** musculoskeletal sarcoma surgery, benchmarking, real-world-time data assessment, risk factors, 30-day morbidity, Clavien-Dindo complications, Charlson Comorbidity index

## Abstract

**Background**: This study investigates the risk factors associated with postoperative complications in musculoskeletal tumor surgeries and evaluates the impact of benchmarking in enhancing surgical outcomes. **Methods**: Conducted at a tertiary referral center, this retrospective analysis included 196 patients who underwent surgeries for various musculoskeletal tumors, ranging from soft tissue to bone sarcomas. Patient and tumor characteristics, along with surgical interventions and outcomes, were comprehensively assessed using the Charlson Comorbidity Index and the Clavien-Dindo classification. **Results**: Key findings indicate that surgical reconstruction, ASA 3 status, bone tumor presence, and the need for multiple erythrocyte transfusions significantly increase postoperative morbidity. Notably, no significant correlation was found between the Charlson Comorbidity Index scores and the occurrence or severity of complications, challenging the utility of this index in predicting short-term surgical outcomes. **Conclusions**: This study highlights the importance of tailored surgical approaches and emphasizes rigorous preoperative assessments to mitigate risks and enhance patient care. Despite its insights, limitations include its retrospective nature and single-center scope, suggesting a need for broader, multicenter studies to generalize findings. Overall, our results underscore the necessity of integrating clinical assessments with benchmarking data to optimize outcomes in the complex field of musculoskeletal tumor surgery.

## 1. Introduction

Musculoskeletal tumor surgeries present unique challenges due to their complexity and the substantial risk of postoperative complications [1]. Identifying and understanding the risk factors associated with these complications is crucial for optimizing patient outcomes and enhancing healthcare efficiency [2]. This study focuses on exploring these risk factors, aiming to contribute to better surgical planning and risk management.

Benchmarking has emerged as a vital practice in healthcare to evaluate and improve patient outcomes [3,4,5]. By comparing practices and outcomes across institutions or between disciplines, benchmarking helps establish best practices and set standards of care, particularly in high-stakes fields like sarcoma surgery [6,7]. In this context, our study aims to establish the standardized use of benchmarking in sarcoma care to understand how different risk factors influence postoperative morbidity.

Furthermore, the complexities of musculoskeletal tumor surgery, encompassing a broad spectrum of tumor types and surgical interventions, necessitate a detailed examination of postoperative risks. Therefore, this study aims to fill gaps in current knowledge by systematically assessing these risks in relation to patient-specific factors such as the Charlson Comorbidity Index and the nature of the tumor (bone vs. soft tissue). Additionally, we employ the Clavien-Dindo classification of complications to provide a standardized framework for evaluating and reporting surgical outcomes, which is crucial for developing effective risk stratification tools [8,9,10,11].

## 2. Materials and Methods

### 2.1. Study Design

This was a retrospective observational study that used real-world-time data from a tertiary referral center (Cantonal Hospital of Lucerne).

This study was designed to encompass the broad spectrum of musculoskeletal tumor surgeries, ranging from minor excisions to major resections across various anatomical locations, including both benign and malignant lesions. This comprehensive approach reflects the clinical reality surgeons face in orthopedic oncology, thereby enhancing the relevance and applicability of our findings to diverse healthcare settings [3].

### 2.2. Tumor Classification and Surgical Interventions

Malignant, benign, and intermediate (locally aggressive or rarely metastasizing) musculoskeletal tumors were included based on pathological assessments and clinical behavior. Each tumor was classified not only by its nature but also by its location and the type of surgical intervention used, according to the WHO 2020 classification [12]. This detailed categorization allows for a precise analysis of outcomes and complications associated with specific surgical treatments [13].

### 2.3. Study Population and Data Collection

This study included a consecutive cohort of patients from a real-world-time data warehouse who underwent surgical procedures for all musculoskeletal tumors presented at a tertiary referral institution over a four-year period (9 January 2018 to 11 April 2022) [3]. The cohort included patients with both soft tissue and bone tumors, encompassing the entire spectrum from benign and intermediate to malignant tumors and sarcomas. All surgeries were performed by an experienced fellowship-trained sarcoma surgeon (BF) with more than 20 years of experience. Comprehensive data were collected from medical records, including patient demographics, tumor characteristics, surgical details, and postoperative outcomes. 

### 2.4. Assessment of Comorbidities

To systematically assess the impact of comorbidities on surgical outcomes, the Charlson Comorbidity Index (CCI) was calculated [8]. The CCI quantifies the severity of comorbid conditions that might alter the risk of mortality, with each condition assigned a specific weight based on its potential influence on mortality risk. This scoring system encompasses a range of conditions, including but not limited to, heart disease, diabetes, and hypertension, thus providing a comprehensive measure of patient health status. Details of the conditions included in the CCI and their respective weights are presented in Table 1 [9,10,11].

### 2.5. Statistical Analysis

Descriptive statistics were used to summarize demographic and clinical characteristics, with categorical variables (e.g., gender, tumor location) presented as frequencies and percentages and continuous variables (e.g., age, tumor size) as means (± standard deviation) or medians (1st quartile [Q1], 3rd quartile [Q3]), based on distribution. Key associations, such as between the Charlson Comorbidity Index and 30-day postoperative morbidity, were visualized using a density plot and analyzed using chi-square or Fisher’s exact tests for categorical variables and Student’s t-test or Mann–Whitney U tests for continuous ones. Complications were classified according to the Clavien-Dindo classification, which stratifies surgical complications by their severity (Table 2). This categorization helps in the standardized reporting of adverse outcomes and facilitates comparison across studies. Multivariable logistic regression was applied to identify independent predictors of complications, adjusting for potential confounders. Statistical significance was set at a *p*-value of less than 0.05, with all analyses conducted in R (version 4.3.1).

## 3. Results

### 3.1. Patient and Tumor Characteristics

This study analyzed 187 patients undergoing 196 musculoskeletal tumor surgeries, encompassing an array of soft tissue and bone lesions. Specifically, 146 patients presented with soft tissue lesions and 41 with bone lesions, classified into 100 malignant, 42 intermediate, and 45 benign types. Noteworthy, a significant subset of soft tissue lesions were lipomatous, with a variety of other types including undifferentiated sarcomas and myo-/fibroblastic tumors. Bone lesions varied from chondrogenic to osteoblastic and included rare types such as Ewing sarcomas and notochordal tumors. Detailed categorizations of these lesions are provided in Table 3.

### 3.2. Demographic Overview

The demographic and clinical characteristics of the analyzed patients, including age, gender, racial composition, and health status, highlight significant variations in patient profiles and medical complexities. Detailed demographic data, stratified by tumor type and presented with associated complication rates, are outlined in Table 4 and Table 5.

### 3.3. Comorbidities and Medication

The study cohort exhibited a range of comorbid conditions, with hematologic diseases, central nervous system disorders, and gastrointestinal issues being the most prevalent. Additional comorbidities included respiratory, cardiovascular, and neuromuscular disorders, along with specific cases of previous childhood malignancies and bleeding disorders (Table 6). This diversity in health conditions highlights the medical complexity of the patient population and underscores the importance of comprehensive preoperative assessment.

### 3.4. Therapy

The treatment modalities for the 187 patients encompassed a combination of radiotherapy, chemotherapy, and surgical interventions. A significant portion of the cohort underwent neoadjuvant treatments, reflecting the complex therapeutic strategies employed in managing musculoskeletal tumors (Table 7). Surgical approaches varied significantly, with the majority of surgeries being elective, highlighting the planned and non-urgent nature of most interventions.

### 3.5. Association between Reconstruction and 30-Day Postoperative Morbidity

This study assessed the impact of surgical reconstruction on postoperative complications. Analysis indicated a notably higher complication rate in patients undergoing reconstruction compared to those without. Specifically, reconstruction surgeries exhibited a 33.7% complication rate versus 16.8% in non-reconstruction cases, demonstrating a statistically significant difference (*p* = 0.008). Notably, the type of tumor and tissue significantly influenced complication rates, with sarcoma and bone tumor patients showing the highest rates post-reconstruction (Table 8).

### 3.6. Complications

The occurrence and nature of complications among the 185 patients and 196 surgeries were analyzed. Complications were observed after 48 (24.5%) surgeries, with a higher incidence in sarcoma patients (29.8%) compared to those with benign tumors (12.9%, *p* = 0.007). Complication rates varied significantly based on the type of treatment and tumor characteristics. Notably, adjuvant therapy—both radiotherapy (57.1%) and chemotherapy (66.6%)—was associated with higher complication rates compared to other treatment modalities.

Complications were more prevalent in patients with bone tumors (38.6%) than in those with soft tissue tumors (20.4%). Additionally, axial tumor location, functional status, and the presence of metastasis influenced complication rates, underscoring the impact of patient and tumor specifics on surgical outcomes. The most common complications included infections, wound dehiscence, and the requirement for blood transfusions or reoperations (Table 9 and Table 10).

### 3.7. Risk Factors of 30-Day Postoperative Morbidity

Our analysis identified several independent risk factors associated with complications within 30 days post-surgery.

Applying a multivariable regression model and after adjusting for the known prognostic factors age and dignity, patients with ASA 3 compared to the ASA 1 category and patients with bone tumors compared to soft tissue tumors were at increased odds of suffering from a complication, OR 3.74 (95% CI; 1.24, 12.0), *p* = 0.02 and OR 2.26 (95% CI; 1.03, 4.93), *p* = 0.04, respectively. Furthermore, the odds of a postoperative complication increased with the number of erythrocyte concentrates administered, OR 2.62 (95% CI; 1.53, 6.06), *p* = 0.005.

Conversely, the Charlson Comorbidity Index (CCI) and patient functional status were not significantly associated with the occurrence of complications. Additionally, neither neoadjuvant radiotherapy nor systemic therapy was linked to a higher rate of postoperative morbidity. This highlights the specific clinical and surgical factors that more directly influence outcomes in our patient cohort (Table 11).

### 3.8. Association between Charlson Comorbidity Index and 30-Day Postoperative Morbidity

**Figure 1:**
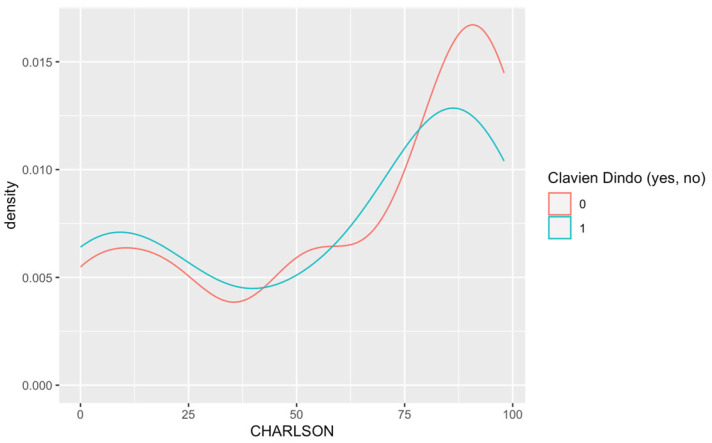
This density plot reveals no association of the Charlson Comorbidity Index with the 30-day postoperative morbidity rate according to Clavien-Dindo.

Our analysis showed that the Charlson Comorbidity Index (CCI) does not significantly predict the occurrence or severity of complications within 30 days post-surgery. Both the occurrence of complications and their severity were not statistically influenced by the CCI, with odds ratios of 1.30 (*p* = 0.14) and 1.09 (*p* = 0.54), respectively. This suggests that the CCI, despite its broad usage in predicting long-term mortality, may not be a reliable indicator of short-term postoperative complications in this patient population (Table 12) (Figure 1).

## 4. Discussion

This study provides a comprehensive evaluation of postoperative outcomes in musculoskeletal tumor surgeries, uncovering significant predictors of surgical morbidity through a detailed analysis. Our results indicate that surgical reconstruction and ASA 3 status are strongly associated with increased complication rates. Furthermore, the bone tumor is identified as a critical factor that elevates postoperative morbidity. This study also underscores the impact of multiple erythrocyte transfusions on increasing morbidity, emphasizing the need for meticulous blood management and surgical planning. These findings advocate for the implementation of specialized risk management strategies, aiming to refine surgical interventions and enhance patient outcomes in the complex landscape of musculoskeletal tumor surgery [3,7,14].

Our examination extends beyond identifying risk factors to analyzing their implications within the surgical context, focusing particularly on reconstructive surgeries, erythrocyte transfusion requirements, and critical patient risk factors. In line with findings from Gallaway et al. and Gonzalez et al. [1,13], our research confirms that reconstructive surgeries significantly elevate postoperative complications, illustrating the complex surgical decisions that impact patient outcomes. Similarly, our emphasis on erythrocyte transfusions as a risk factor for increased morbidity parallels observations from Gonzalez et al. [1], who noted complications related to low hematocrit levels, suggesting a shared concern across studies about blood management in surgical procedures. Our analysis further aligns with Gallaway et al. [13] regarding the critical influence of bone tumor presence on postoperative outcomes, affirming that these inherent patient conditions significantly contribute to morbidity, consistent with their reported findings. However, a unique aspect of our study is the detailed exploration of ASA 3 status as a modifiable preoperative assessment parameter, which has been less emphasized in similar studies but is crucial for tailoring surgical and anesthetic strategies to mitigate risks.

Despite these similarities, our study also highlights a gap in the literature regarding standardized approaches to measuring and reporting these risk factors, especially compared to the uniform assessment methods evident in studies like those by Gallaway et al. and Gonzalez et al. [1,13]. Conversely, Hoftiezer et al., analyzing factors associated with 30-day morbidity following upper extremity surgery, did not associate findings from above but linked an increased BMI, increased operative time, flap reconstruction, and tumor size > 5 cm with 30-day postoperative complications [15]. This disparity regarding parameters to be analyzed and findings underscores the necessity for more consistent and comprehensive methodologies to assess and report 30-day morbidity in musculoskeletal tumor surgery research, fostering better comparability and reliability of outcomes across studies.

The key insights derived from our analysis of 30-day postoperative morbidity in musculoskeletal tumor surgeries emphasize the need for refined surgical protocols and robust preoperative assessments. Given the heightened risks associated with ASA 3 status, bone tumors, and reconstructive surgeries, personalized surgical planning becomes imperative. Our findings suggest that such plans should incorporate targeted preoperative assessments and tailored intraoperative strategies to effectively manage these identified risks. Additionally, the significant role of erythrocyte transfusions in elevating morbidity rates calls for the development of stringent blood management protocols, potentially including preoperative optimization of hemoglobin levels and judicious intraoperative transfusion practices [16]. Future research should focus on enhancing predictive models for postoperative complications by integrating these and other clinical variables. This would not only refine the accuracy of risk assessments but also aid in developing specific interventional strategies aimed at minimizing complications. Prospective multicenter studies are necessary to validate our findings and to explore the effectiveness of these tailored approaches in diverse surgical settings. Moreover, our results underscore the importance of considering both inherent and modifiable risk factors in the surgical management of musculoskeletal tumors, suggesting a multidimensional approach to patient care that adapts to the specificities of each case. These comprehensive efforts will not only advance our understanding of the complex dynamics in musculoskeletal tumor surgeries but also lead to more effective and personalized treatment plans that significantly improve patient outcomes, aligning with the evolving standards of care in the field [6,14,17].

Our study, while providing insightful observations on musculoskeletal tumor surgeries, is not without limitations. The retrospective nature of our data collection introduces inherent biases, including potential selection biases and limitations in data completeness that may affect the findings’ generalizability. Moreover, this study’s focus on a single tertiary care center may not fully represent the wider patient population typically encountered in other settings, which could influence the applicability of our results to different healthcare environments. Additionally, the specificity of the patient demographics and tumor types included may limit the ability to generalize our conclusions universally. Future studies could address these limitations by incorporating a multicenter approach and a more diverse patient cohort to enhance the robustness and applicability of the findings.

## 5. Conclusions

In conclusion, this study highlights the importance of tailored surgical planning and rigorous preoperative assessments in reducing postoperative morbidity in musculoskeletal tumor surgeries. Key findings reveal that surgical reconstruction, ASA 3 status, bone tumor presence, and erythrocyte transfusions significantly influence outcomes, emphasizing the need for personalized treatment strategies. Despite its insights, this study’s limitations underscore the necessity for broader, multicenter studies to confirm these findings and refine risk stratification methods. Future research should focus on developing predictive models that integrate clinical and benchmarking data to optimize surgical outcomes in this complex field.

## Figures and Tables

**Table 1:** Charlson Comorbidity Index scoring system [8].

Condition	Weight
Myocardial infection	1
Congestive Heart Failure	1
Peripheral Vascular Disease	1
Cerebrovascular Disease	1
Dementia	1
Chronic Pulmonary Disease	1
Rheumatic Disease	1
Peptic Ulcer Disease	1
Mild Liver Disease	1
Diabetes without Complications	1
Diabetes with Complications	2
Hemiplegia or Paraplegia	2
Renal Disease	2
Any Malignancy	2
Moderate or Severe Liver Disease	3
Metastatic Solid Tumor	6
AIDS/HIV	6

Overview of the Charlson Comorbidity Index (CCI) showing each condition considered in the index and the associated weights used to assess overall comorbidity and predict mortality risk in patients.

**Table 2:** Clavien-Dindo classification [9,10].

I	Any deviation from the normal postoperative course without the need for pharmacological treatment re surgical, endoscopic, and radiological interventions. Allowed therapeutic regimens are drugs as antiemetics, antipyretics, analgesics, diuretics, electrolytes, and physiotherapy. Also included is wound infection opened at the bedside.
II	Requiring pharmacological treatment with drugs other than those allowed for grade I complications. Also included are blood transfusions and total parenteral nutrition.
IIIa	Requiring surgical, endoscopic, or radiological intervention.
IIIb	Intervention under general anesthesia.
IVa	Life-threatening complication (including CNS complications) requiring ICU management; single organ dysfunction.
IVb	Multiorgan dysfunction.
V	Death of patient

**Table 3:** Patient and tumor characteristics.

Category	Total Number	Malignant	Intermediate *	Benign	Specific Types (Number)
Total Patients	187	100	42	45	
Soft Tissue Lesions	146	76	34	36	Lipomatous (64), Undifferentiated Sarcomas (18), Myo-/Fibroblastic (27), Uncertain Origin (14), Smooth Muscle (9), Nerve Sheath (4), Fibrohistiocytic (3)
Bone Lesions	41	24	8	9	Chondrogenic (14), Osteoblastic (12), Ewing Sarcomas (3), Giant-cell rich (2), giant cell tumor (2)

This table Overview of patient demographics and tumor characteristics, detailing the distribution and classification of lesions among the 187 patients studied. The table breaks down the total number of patients presenting with soft tissue and bone lesions, their categorization into malignant, intermediate, and benign types, and details specific tumor types within each category. (* locally aggressive or rarely metastasizing according to WHO 2020).

**Table 4:** Demographic and tumor characteristics stratified by dignity.

Category	Total (*n* = 187)	Sarcoma (*n* = 129)	Benign Tumor (*n* = 58)
**Age [years] median (Q1, Q3)**	57 (42, 67)	58 (46, 70)	50 (37, 63)
**Female**	86 (46%)	58 (45%)	28 (48%)
**Caucasian**	185 (99%)	127 (98%)	58 (100%)
**Functional Status**			
Independent	137 (92%)	86 (91%)	51 (94%)
Partially Independent	10 (6.7%)	9 (9.5%)	1 (1.9%)
Dependent	2 (1.3%)	0 (0.0%)	2 (3.7%)
Unknown	36 (19.3%)	34 (26.4%)	4 (6.9%)
**ASA Status**			
1	41 (22%)	20 (16%)	21 (36%)
2	115 (61%)	82 (64%)	33 (57%)
3	31 (17%)	27 (21%)	4 (6.9%)
**Current Smoker**	24 (13%)	13 (10%)	11 (20%)
**Disseminated Cancer**	7 (3.7%)	5 (3.9%)	0 (0.0%)
**Tumor Location**			
Appendicular	148 (79%)	102 (79%)	46 (79%)
Axial	39 (21%)	27 (21%)	12 (21%)
**Tumor Type**			
Bone	41 (22%)	26 (20%)	15 (26%)
Soft Tissue	146 (78%)	103 (80%)	43 (74%)
**Charlson Comorbidity score > 5**	24 (13%)	22 (17%)	2 (3.4%)

This table summarizes demographic and clinical characteristics of the study population, stratified by tumor classification. Age is presented as a median with first (Q1) and third quartiles (Q3). Categorical variables are presented as n (%). Complications were captured using the Clavien-Dindo classification.

**Table 5:** Complications stratified by dignity.

Category	Total (*n* = 196)	Sarcoma (*n* = 134)	Benign Tumor (*n* = 62)
**Complications according to Clavien-Dindo**			
No complications	148 (75.5%)	94 (70.1%)	54 (87.1%)
Grade 1	25 (12.8%)	20 (14.9%)	5 (8.1%)
Grade 2	13 (6.6%)	13 (9.7%)	0 (0%)
Grade 3	10 (5.1%)	7 (5.2%)	3 (4.8%)
**Presence of complication**	48 (24.5%)	40 (29.8%)	8 (12.9%)

This table summarizes the complications according to Clavien-Dindo stratified by dignity. Categorical variables are presented as n (%).

**Table 6:** Overview of comorbidities details the prevalence rates of these conditions, providing a clear picture of the health challenges faced by the patient cohort.

Comorbidity	Number of Patients (%)
Hematologic Disease	21 (11.2%)
Central Nervous System Disorder	20 (10.7%)
Gastrointestinal Disease	17 (9.1%)
Chronic Obstructive Pulmonary Disease	1 (0.5%)
History of Other Pulmonary Disease	7 (3.7%)
Congestive Heart Failure	1 (0.5%)
Neuromuscular Disorder	1 (0.5%)
Previous Childhood Malignancy	2 (1.1%)
Bleeding Disorder	2 (1.1%)
Diabetes mellitus	6 (3.2%)

Overview of the prevalence of various comorbidities among the 187 patients studied. Data are presented as the number (%) of included patients.

**Table 7:** Treatment modalities and surgery type for study population.

Treatment Type	Number of Sarcoma Patients	Percentage (%)
Neoadjuvant Radiotherapy	64	32.7
Adjuvant Radiotherapy	7	3.6
Neoadjuvant Chemotherapy	18	9.2
Neoadjuvant and Adjuvant Chemotherapy	3	1.5

This table Summary of treatment modalities and types of surgery. The table details the number and percentage of sarcoma patients that received various types of radiotherapy and chemotherapy.

**Table 8:** Association between reconstruction and 30-day postoperative morbidity.

Patient Group	Surgeries with Reconstruction *n* = 89	*p*-Value	Surgeries without Reconstruction *n* = 107
**Overall Complication Rate**	30/89 (33.7%)	0.008 *	18/107 (16.3%)
**Complication Rate by Tumor**			
Sarcoma	27/79 (34.2%)	0.98 **	13/55 (23.6%)
Benign Tumor	3/10 (30%)		5/52 (9.6%)
**Complication Rate by Tissue Type**			
Bone Tumor	13/27 (48.2%)	0.09 **	4/17 (23.5%)
Soft Tissue Tumor	17/62 (27.4%)		14/90 (15.6%)

This table details the association between surgical reconstruction and 30-day postoperative morbidity, comparing complication rates among patients undergoing reconstruction versus those who did not, further stratified by tumor and tissue type. Statistical significance is noted where applicable. * comparing patients with reconstruction versus patients without; ** comparing benign with sarcoma or bone tumor with soft tissue tumors.

**Table 9:** Summary of postoperative complications and associated parameters.

Category	Total (*n* = 196)	Complication (*n* = 89)	No Complication (*n* = 107)	*p*-Value
**Age [years]** **(median (Q1, Q3))**	56 (42, 67)	60 (48, 69)	51 (38, 63)	0.01
**Female**	86 (46%)	44 (49%)	47 (44%)	0.40
**Caucasian**	185 (99%)	89 (100%)	105 (98%)	0.93
**Functional Status**				0.002
Independent	137 (92%)	56 (62.9%)	89 (83.1%)	
Partially Independent	10 (6.7%)	9 (10.1%)	1 (0.9%)	
Dependent	2 (1.3%)	2 (2.2%)	1 (0.9%)	
Unknown	36 (19.3%)	22 (24.7%)	16 (15.1%)	
**ASA Status**				<0.001
1	41 (22%)	10 (11%)	33 (31%)	
2	115 (61%)	54 (61%)	65 (61%)	
3	31 (17%)	25 (28%)	9 (8.4%)	
**Current Smoker**	24 (13%)	8 (9.0%)	17 (16%)	0.14
**Disseminated Cancer**	7 (3.7%)	6 (6.7%)	5 (4.7%)	0.60
**Tumor Location**				0.44
Appendicular	148 (79%)	65 (75%)	87 (81%)	
Axial	39 (21%)	22 (25%)	20 (19%)	
**Tumor Type**				0.33
Bone	41 (22%)	27 (30%)	17 (16%)	
Soft Tissue	146 (78%)	62 (70%)	90 (84%)	
**Charlson Comorbidity** **Score > 5**	24 (13%)	18 (20%)	11 (10%)	0.05
**Radiotherapy** None Neoadjuvant Adjuvant	(*n* = 187) * 118 (63%) 64 (34%) 5 (2.7%)	(*n* = 85) * 39 (46%) 42 (49%) 4 (4.7%)	(*n* = 102) * 79 (77%) 22 (22%) 1 (1.0%)	<0.001
**Chemotherapy** None Neoadjuvant Adjuvant Sandwich	(*n* = 187) * 164 (88%) 17 (9.1%) 3 (1.6%) 3 (1.5%)	(*n* = 85) * 69 (81%) 13 (15%) 2 (2.4%) 1 (1.2%)	(*n* = 102) * 95 (93%) 4 (3.9%) 1 (1.0%) 2 (2.1%)	0.01

* Numbers for radiotherapy and chemotherapy are based on the number of patients and not on the number of surgeries performed. This table provides a comprehensive overview of the postoperative complications observed in the study cohort, broken down by various categories including tumor type, treatment modality, demographic factors, functional status, and specific complication types.

**Table 10:** Summary of the types of infections.

Type of Infection	Number	Percentage (%)
**Surgical Site Infections**	8/196	4.1
**Pneumonia**	2/196	1.0
**Sepsis**	2/196	1.0
**Wound Dehiscence (Deep)**	7 /196	3.6
**Wound Dehiscence (Superficial)**	7/196	3.6
**Venous Thromboembolism**	4/196	2.0
**Allograft Failure**	1/196	0.5
**Prosthesis Failure**	1/196	1.0
**Flap Failure**	1/196	0.5
**Blood Transfusion**	17/196	8.7
**Reoperation**	10/196	5.1
**Re-Admission**	6/196	3.1

**Table 11:** Univariable logistic regression analysis for 30-day postoperative complications.

Variable	Odds Ratio (95% CI)	*p*-Value
**Age**	1.0 (0.98, 1.01)	0.06
**Gender** (Male vs. Female)	1.15 (0.60, 2.24)	0.71
**Tumor Type**		
-Bone (vs. Soft Tissue)	2.46 (1.18, 5.06)	0.02
**Presence of Metastasis**	4.09 (1.18, 14.8)	0.03
**Tumor Classification**		
-Sarcoma (vs. benign)	2.87 (1.31, 7.02)	0.01
**Radiotherapy**		
-Neoadjuvant (vs. adjuvant vs. none)	1.18 (0.59, 2.32)	0.60
**Systemic Therapy**		
-Neoadjuvant (vs. adjuvant vs. none)	2.27 (0.84, 5.87)	0.01
**ASA Status**		
-ASA 2 (vs. ASA 1)	1.05 (0.44, 2.69)	0.92
-ASA 3 (vs. ASA 1)	4.38 (1.62, 12.7)	0.01
**Charlson Comorbidity Index (CCI)**	1.13 (0.96, 1.33)	0.14
**Functional Status**	2.29 (0.91, 5.85)	0.07
**Number of Erythrocyte Concentrations**	3.00 (1.67, 7.24)	0.01

This table presents the results of univariable regression analyses assessing the risk factors for 30-day postoperative morbidity.

**Table 12:** Association of Charlson Comorbidity Score with 30-day postoperative morbidity.

Outcome	Odds Ratio (OR)	95% Confidence Interval (CI)	*p*-Value
Occurrence of Complications	1.13	0.96,1.33	0.14
Complication Severity	1.09	0.83–1.43	0.54

This table shows the results of a univariable logistic regression with the occurrence of complications as the dependent variable and the results of a proportional odds model with the complication severity as graded by Clavien-Dindo as the dependent variable.

## Data Availability

The original contributions presented in the study are included in the article material, further inquiries can be directed to the corresponding author.
